# ChemBounce: a computational framework for scaffold hopping in drug discovery

**DOI:** 10.1093/bioinformatics/btaf501

**Published:** 2025-09-11

**Authors:** Woo Dae Jang, Changdai Gu, Yumi Noh, Kwang-Seok Oh, Jae Yong Ryu

**Affiliations:** Data Convergence Drug Research Center, Korea Research Institute of Chemical Technology, Daejeon 34114, Republic of Korea; Department of Medicinal and Pharmaceutical Chemistry, University of Science and Technology, Daejeon 34129, Republic of Korea; Artificial Intelligence Laboratory, Oncocross Co. Ltd., Seoul 05836, Republic of Korea; Department of Artificial Intelligence, School of Computing, Yonsei University, Seoul 03722, Republic of Korea; Medical Research Center, College of Medicine, Yonsei University, Seoul 03722, Republic of Korea; Data Convergence Drug Research Center, Korea Research Institute of Chemical Technology, Daejeon 34114, Republic of Korea; Department of Medicinal and Pharmaceutical Chemistry, University of Science and Technology, Daejeon 34129, Republic of Korea; Data Convergence Drug Research Center, Korea Research Institute of Chemical Technology, Daejeon 34114, Republic of Korea; Department of Medicinal and Pharmaceutical Chemistry, University of Science and Technology, Daejeon 34129, Republic of Korea; School of Systems Biomedical Science, Soongsil University, Seoul 06978, Republic of Korea

## Abstract

**Summary:**

Scaffold hopping is a critical strategy in medicinal chemistry for generating novel and patentable drug candidates. Here, we present ChemBounce, a computational framework designed to facilitate scaffold hopping by generating structurally diverse scaffolds with high synthetic accessibility. Given a user-supplied molecule in SMILES format, ChemBounce identifies the core scaffolds and replaces them using a curated in-house library of over 3 million fragments derived from the ChEMBL database. The generated compounds are evaluated based on Tanimoto and electron shape similarities to ensure retention of pharmacophores and potential biological activity. By enabling systematic exploration of unexplored chemical space, ChemBounce represents a valuable tool for hit expansion and lead optimization in modern drug discovery.

**Availability and implementation:**

The source code for ChemBounce is available at https://github.com/jyryu3161/chembounce. In addition, a cloud-based implementation of ChemBounce is available as a Google Colaboratory notebook.

## 1 Introduction

Scaffold hopping, a term first coined by Schneider and colleagues in 1999, has become an integral approach in medicinal chemistry and drug discovery (Schneider *et al.* 1999). Originating from computational chemistry and virtual screening techniques, it aims to identify compounds with different structures but similar biological activities or property profiles (Hu *et al.* 2017). This approach helps overcome the challenges of drug discovery, such as intellectual property constraints, poor physicochemical properties, metabolic instability, and toxicity issues. In fact, scaffold hopping has led to the successful development of marketed drugs, such as Vadadustat, Bosutinib, Sorafenib, and Nirmatrelvir (Acharya *et al.* 2024). While the modification or substitution of core molecular structures may be conducted based on existing chemical knowledge, computational frameworks often enable more extensive scaffold hopping, which involves generating unexpected molecules from existing knowledge (Gong *et al.* 2013, Zheng *et al.* 2021).

Various computational methods have been developed for scaffold hopping, including those based on pharmacophore models, shape similarity, alignment-independent 3D or connectivity descriptors, and fragment-based approaches (Schuffenhauer 2012). Of these, approaches based on the pharmacophore concept have traditionally been favored. Pharmacophore-based scaffold hopping strategies involve replacing scaffolds under conditions where functional groups critical to interaction with the target are retained. Although there is an inherent connection between pharmacology and scaffold hopping, alternative computational techniques have been successfully applied as well. For instance, shape-based searching presents a practical approach for scaffold hopping (Rush *et al.* 2005). Furthermore, machine and deep learning algorithms, such as self-organizing maps for visualizing molecular distributions and transformer, have shown applicability in this area (Schneider *et al.* 2009, Zheng *et al.* 2021). Existing computational tools have a limited number of algorithms compared to the variety of approaches used in scaffold hopping, and few open-source packages are available. In this study, we developed an open-source tool for scaffold hopping that considers both synthetic accessibility and Tanimoto and electron shape similarities (Armstrong *et al.* 2010).

## 2 Description

**Figure 1.**
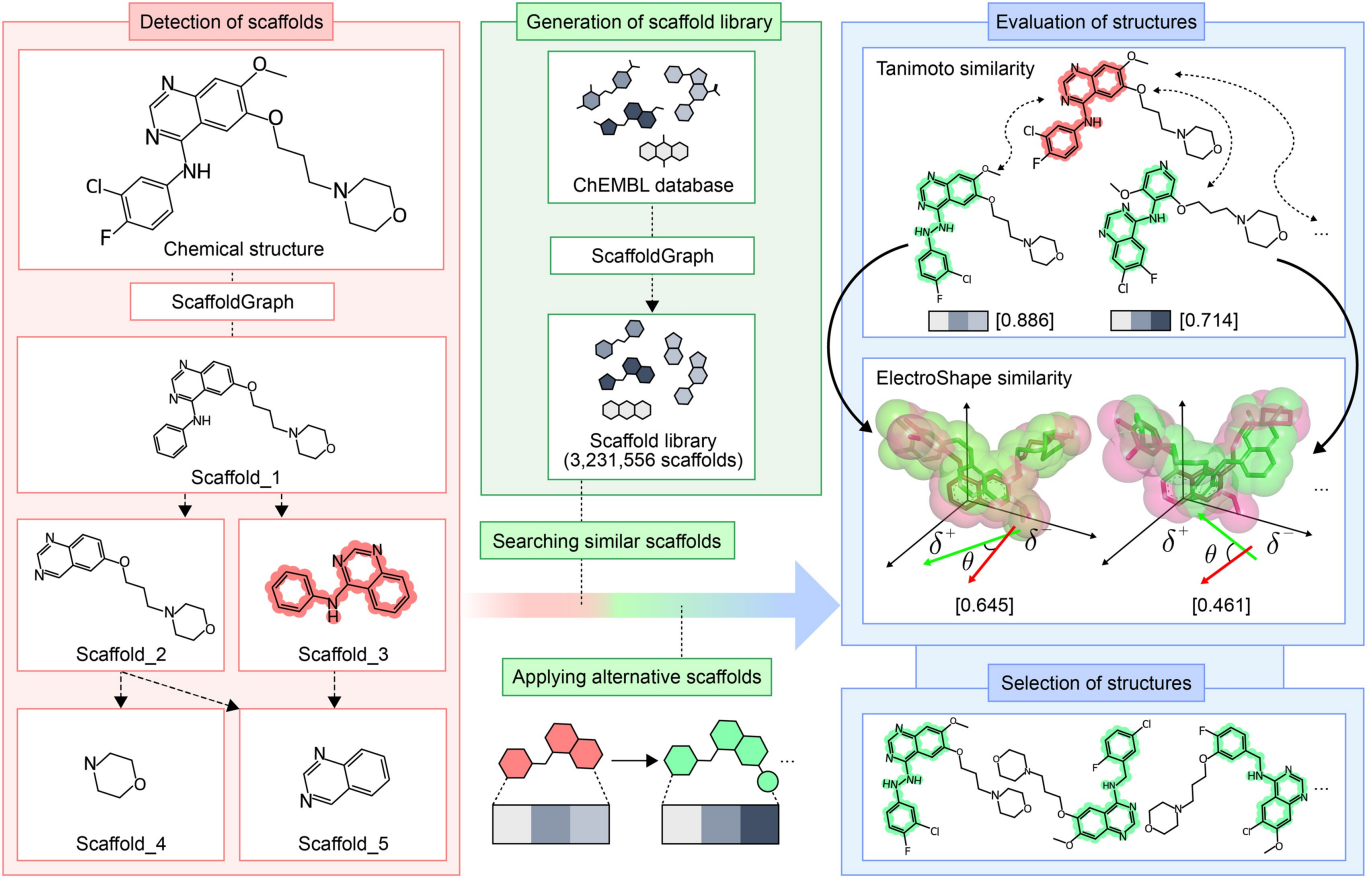
The workflow of scaffold hopping by ChemBounce. A SMILES string of an active compound is received as the input. Fragmentation of the input structure is performed to identify possible scaffolds. Scaffold similarity is calculated by ECFP4. The scaffold library with about 3.2 million compounds is generated from ChEMBL. Similar scaffolds are replaced with detected scaffolds of the input structure. To preserve the biological activity of the input structure by retaining its pharmacophore, electron shape similarity is calculated between the designed molecules and the input structure.

ChemBounce is a computational tool designed to efficiently generate novel chemical structures while preserving the biological activity of the original structure by applying shape-based similarity constraints to the input structure (Fig. 1). It leverages a diverse scaffold library curated from the ChEMBL database, consisting of synthesis-validated fragments, thereby ensuring that the generated structures exhibit high synthetic accessibility.

### 2.1 Workflow

ChemBounce initiates its process by receiving the input structure as a SMILES string, which is then fragmented to identify the diverse scaffold structures included in the input structure. All fragments are generated by applying a set of rules to specify the bonds to break based on the graph analysis algorithm using the ScaffoldGraph (Scott and Chan 2020). We used the HierS methodology (Wilkens *et al.* 2005) among the scaffold building algorithms that comprise ScaffoldGraph to generate scaffolds. HierS algorithm decomposes molecules into ring systems, side chains, and linkers, where atoms external to rings with bond orders >1 and double-bonded linker atoms are preserved within their respective structural components. Basis scaffolds are generated by removing all linkers and side chains, while superscaffolds retain linker connectivity. The recursive process systematically removes each ring system to generate all possible combinations until no smaller scaffolds exist (Fig. 1, available as supplementary data at *Bioinformatics* online).

To establish a comprehensive reference dataset, we constructed an in-house scaffold library from the ChEMBL database containing 3 231 556 unique scaffolds. This library was generated by applying the HierS algorithm to the entire ChEMBL compound collection, systematically decomposing each molecule to identify all possible ring system combinations through recursive fragmentation. The resulting scaffolds underwent rigorous deduplication to eliminate redundant structures, ensuring each scaffold represents a unique structural motif. Following the HierS methodology, single benzene rings were excluded from the basis scaffold library due to their ubiquitous presence in natural compounds and limited discriminating value for meaningful scaffold hopping applications.

A molecule can have multiple scaffolds, of which one specific scaffold can be used as a query scaffold. Scaffolds similar to the query scaffold are identified from this curated library through Tanimoto similarity calculations based on molecular fingerprints. New molecules are generated by replacing the query scaffold with candidate scaffolds from the library. To maintain the biological activity of the input structure, the various molecular structures generated are subjected to a rescreening process where only compounds with similar pharmacophores through Tanimoto and electron shape similarities are selected. We computed the electron shape similarity of the compounds using the ElectroShape (Armstrong *et al.* 2010) in the ODDT (Wójcikowski *et al.* 2015) Python library.

### 2.2 Usage

The command line below is an example of how to use the ChemBounce program.

python  chembounce.py  −o  OUTPUT_DIRECTORY  −i

  INPUT_SMILES  −n  NUMBER_OF_STRUCTURES  −t

  SIMILARITY_THRESHOLD

where OUTPUT_DIRECTORY is the location of output directory. The INPUT_SMILES is a text containing the small molecules in SMILES format. The -n parameter controls the number of structures for each fragment to generate by scaffold hopping. The -t parameter allows the user to specify the Tanimoto similarity threshold between INPUT_SMILES and the generated SMILES (default is 0.5). Additionally, ChemBounce provides users with the flexibility to retain specific substructures of interest during the scaffold hopping process, enabling tailored molecular design when particular motifs must be conserved for biological activity. This functionality is implemented through the - -core_smiles option, which allows the user to specify which molecular fragments to maintain unchanged during scaffold replacement. Users can constrain the search space to preserve critical pharmacophoric elements while exploring structural diversity in non-essential regions, ensuring that scaffold hopping occurs only in designated areas of the input structure.

For advanced users requiring custom scaffold libraries, ChemBounce supports the - -replace_scaffold_files option, which enables the platform to operate with user-defined scaffold sets instead of the default ChEMBL-derived library. This functionality allows researchers to incorporate domain-specific or proprietary scaffold collections tailored to their particular research objectives, such as natural product-focused libraries or synthetic building block databases. Users can specify their custom scaffold library by providing appropriately formatted scaffold files, enabling ChemBounce to perform scaffold hopping within the constraints of their specialized chemical space.

ChemBounce requires valid SMILES strings for proper scaffold analysis. Common input failures include invalid atomic symbols not present in the periodic table, incorrect valence assignments violating standard bonding rules, and salt or complex forms containing multiple components separated by “.” notation. SMILES strings with malformed syntax such as unbalanced brackets, invalid ring closure numbers, or incorrect stereochemistry will generate parsing errors. Users should preprocess multi-component systems to extract the primary active compound and validate SMILES strings using standard cheminformatics tools prior to analysis. When invalid inputs are encountered, ChemBounce provides detailed error messages with specific remediation strategies. A comprehensive failure-case reference sheet is available in Table 1, available as supplementary data at *Bioinformatics* online.

### 2.3 Key features

ChemBounce addresses critical limitations in current scaffold hopping methodologies through three distinctive features. First, the framework uses a curated scaffold library derived from synthesis-validated ChEMBL fragments, ensuring that generated compounds possess practical synthetic accessibility. Second, we implemented ElectroShape-based molecular similarity calculation that considers both charge distribution and 3D shape properties, ensuring that scaffold-hopped compounds maintain structural compatibility with query molecules and preserve biological activity potential (Armstrong *et al.* 2010). Third, ChemBounce provides comprehensive open-source accessibility through GitHub (https://github.com/jyryu3161/chembounce) and cloud-based execution via Google Colaboratory, eliminating installation barriers for experimental chemists (Fig. 2, available as supplementary data at *Bioinformatics* online).

Performance validation was conducted across diverse types of molecules, including peptides (Kyprolis, Trofinetide, Mounjaro), Macrocyclic compounds (Pasireotide, Motixafortide), and small molecules (Celecoxib, Rimonabant, Lapatinib, Trametinib, Venetoclax) with molecular weights ranging from 315 to 4813 Da. Processing times varied from 4 s for smaller compounds to 21 min for complex structures, demonstrating scalability across different compound classes (Table 2, available as supplementary data at *Bioinformatics* online).

Additionally, the performance of ChemBounce was evaluated by comparison with several commercial scaffold hopping tools to validate its practical utility. Comparative analyses were conducted using five approved drugs—losartan, gefitinib, fostamatinib, darunavir, and ritonavir—against five established platforms: Schrödinger’s Ligand-Based Core Hopping and Isosteric Matching, and BioSolveIT’s FTrees, SpaceMACS, and SpaceLight. Key molecular properties of the generated compounds, including SAscore, QED, molecular weight, LogP, number of hydrogen bond donors and acceptors, and the synthetic realism score (P_Real_) from AnoChem (Gu *et al.* 2024), were assessed (Figs 3–7, available as supplementary data at *Bioinformatics* online). In addition, the performance of ChemBounce was profiled under varying internal parameters, including the number of fragment candidates (1000 versus 10 000), Tanimoto similarity thresholds (0.5 versus 0.7), and the application of Lipinski’s rule of five filters (Figs 8–12, available as supplementary data at *Bioinformatics* online). Overall, ChemBounce tended to generate structures with lower SAscores, indicating higher synthetic accessibility, and higher QED values, reflecting more favorable drug-likeness profiles compared to existing scaffold hopping tools.

## 3 Conclusion

We developed a standalone open-source tool, ChemBounce, which predicts chemically novel compounds starting from known active compounds by modifying the central core structure of the molecule. ChemBounce could generate novel compounds with remaining pharmacophores of the input chemical structure and high synthetic accessibility. ChemBounce is expected to improve the efficiency of molecule design by scaffold hopping.

## Supplementary Material

btaf501_Supplementary_Data

## Data Availability

The source code and the data required to run the ChemBounce program are available at https://github.com/jyryu3161/chembounce.
