# Supplementary material for: ChemBounce: a computational framework for scaffold hopping in drug discovery
Source: Bioinformatics. 2025 Sep 11;41(9):btaf501. doi: 10.1093/bioinformatics/btaf501 (PMC12449259; doi:10.1093/bioinformatics/btaf501)
Supplement: btaf501_Supplementary_Data [file btaf501_supplementary_data.docx]

Supplementary data

ChemBounce: a computational framework for scaffold hopping in drug discovery

Woo Dae Jang^1,2,†^, Changdai Gu^3,4,5,†^, Yumi Noh^1,2,^, Kwang-Seok Oh^1,2^ and Jae Yong Ryu^6,*^

^1^Data Convergence Drug Research Center, Korea Research Institute of Chemical Technology, 141 Gajeong-ro, Yuseong-gu, Daejeon, 34114, Republic of Korea. ^2^Department of Medicinal and Pharmaceutical Chemistry, University of Science and Technology, Daejeon 34129, Republic of Korea. ^3^Artificial Intelligence Laboratory, Oncocross Co., Ltd., 7, Beobwon-ro 11-gil, Songpa-gu, Seoul 05836, Republic of Korea. ^4^Department of Artificial Intelligence, College of Computing, Yonsei University, 50 Yonsei-ro, Seodaemun-gu, Seoul, 03722, Republic of Korea. ^5^Medical Research Center, College of Medicine, Yonsei University, 50 Yonsei-ro, Seodaemun-gu, Seoul 03722, Republic of Korea. ^6^School of Systems Biomedical Science, Soongsil University, 369 Sangdo-Ro, Dongjak-Gu, Seoul 06978, Republic of Korea.

*To whom correspondence should be addressed.

†These authors contributed equally.

**Contents**

**A. Scaffold fragmentation algorithm (Figure S1) Page 2**

**B. Google Colaboratory notebook usage (Figure S2) Page 3**

**C. Baseline platforms (Schrödinger) Page 4**

**D. Baseline platforms (BioSolveIT) Page 4-5**

**E. Performance indexes Page 5-6**

**F. Comparative analysis of molecular properties in scaffold hopping Page 6-19**

- **Molecular properties of scaffold-hopping results for baseline platforms and ChemBounce for approved drugs (Figures S3-S7) Page 7-13**
- **Molecular properties of scaffold-hopping results for approved drugs under various ChemBounce conditions (Figures S8-S12) Page 14-19**

**G. A comprehensive failure-case reference sheet (Table S1) Page 20**

**H. Performance validation of ChemBounce across diverse molecular types (Table S2) Page 21-22**

**References Page 23**

**A. Scaffold fragmentation algorithm**

**
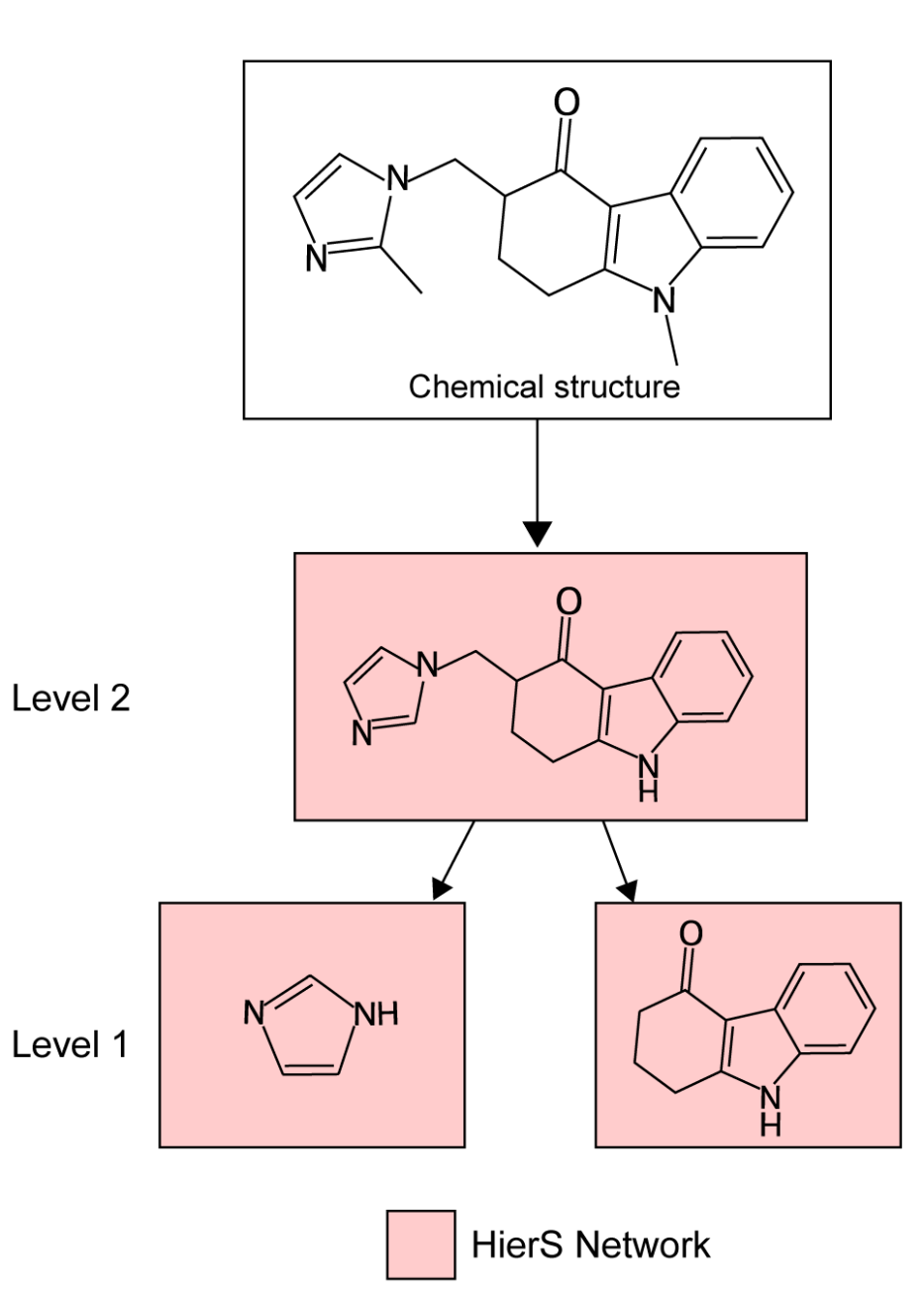
**

**Figure S1. HierS network decomposition of a representative chemical structure.** The hierarchical scaffold fragmentation process demonstrates the systematic breakdown of a complex molecule into constituent scaffold components following the HierS algorithm (Wilkens *et al.*, 2005). The original chemical structure (top) contains three distinct ring systems: an imidazole ring, a cyclohexanone ring, and an indole ring connected through linker atoms. The decomposition follows specific bond-cleavage criteria where atoms external to rings but bonded with bond orders >1 are retained as part of the ring system, and atoms double-bonded to linkers (e.g., the carbonyl group) are preserved within the linker structure. The superscaffold (Level 2) is generated by removing only the side chains while preserving core ring-linker connectivity. Basis scaffolds (Level 1) represent individual ring systems after complete removal of all linkers and chains, where ring systems are defined as one or more rings sharing internal bonds. The imidazole scaffold (left, Level 1) and the indole-cyclohexanone scaffold (right, Level 1) exemplify how fused ring systems are maintained as coherent structural entities. The recursive algorithm generates all possible ring system combinations until no smaller scaffolds exist, with redundant scaffolds (single membership belonging to other classes) automatically removed. Pink boxes indicate scaffolds generated through the HierS network approach.

**B. Google Colaboratory notebook usage**


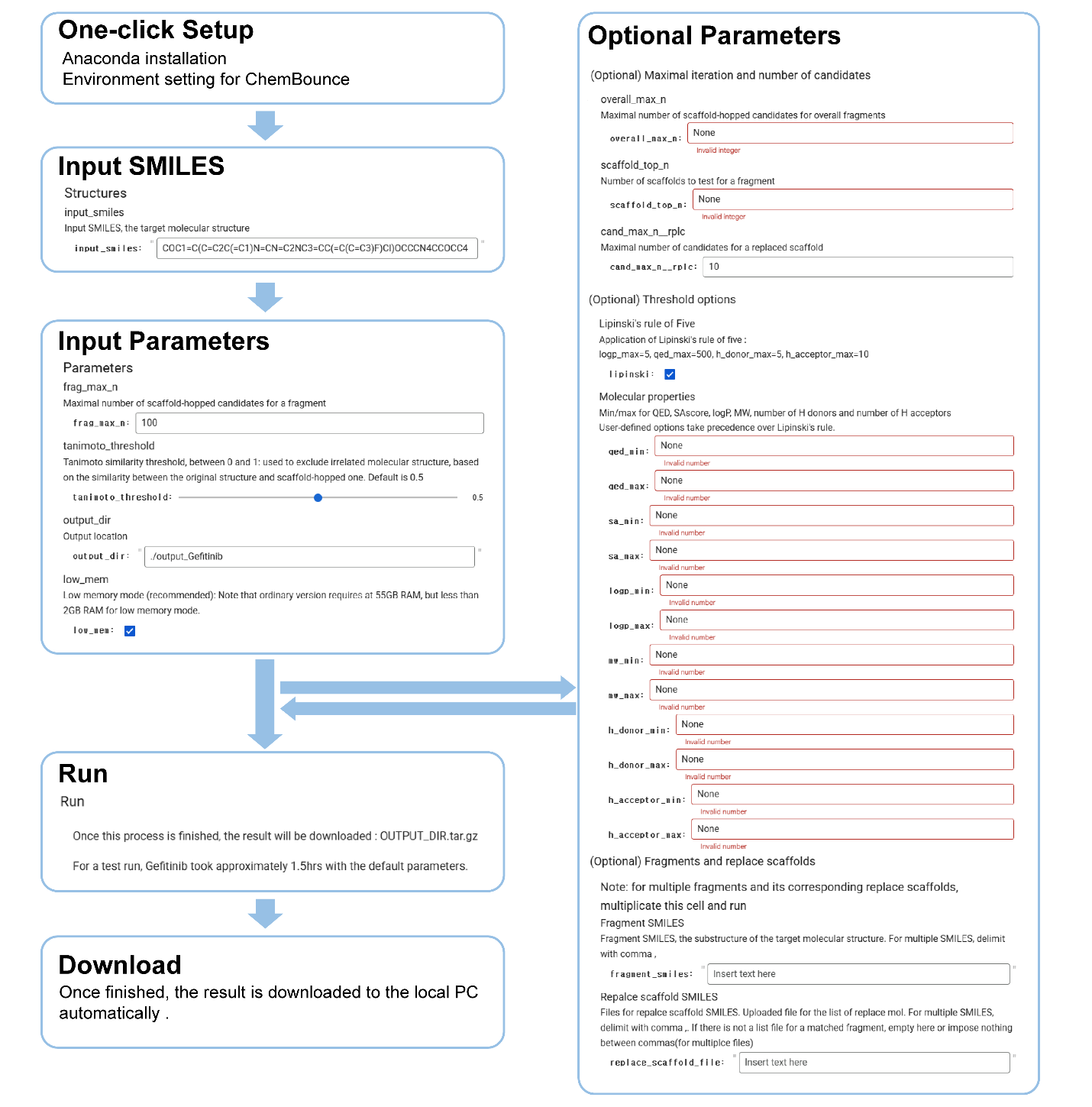


**Figure S2. ChemBounce: Scaffold hopping via Google Colaboratory notebook.** An example of using ChemBounce within Google Colaboratory, a free cloud-based Jupyter notebook environment. This setup enables users to perform scaffold hopping tasks without the need for local installation or advanced configuration. ChemBounce is accessible directly through Colaboratory, streamlining AI-assisted molecular design and scaffold hopping processes for researchers in drug discovery (access the Google Colaboratory notebook through GitHub, <https://github.com/jyryu3161/chembounce>).

**C. Baseline platforms (Schrödinger)**

To benchmark the performance of ChemBounce, we compared it with two scaffold hopping tools available in the Schrödinger suite: Ligand-Based Core Hopping and Isosteric Matching. Both modules aim to identify alternative scaffolds for a given template ligand by: (i) defining a scaffold, (ii) replacing it with candidates from a scaffold library, and (iii) scoring the resulting ligands based on their similarity to the original template.

Ligand-Based Core Hopping primarily evaluates the shape similarity between the modified and template ligands, with an optional feature to enforce hydrogen bond constraints. In contrast, Isosteric Matching additionally allows shape-based optimization to accommodate protein binding site geometry and can restrict ligand penetration into defined spatial regions.

For scaffold definition, we employed the BRICS algorithm, which fragments molecules at synthetically accessible bonds (Degen *et al.*, 2008). The alternative scaffold candidates were sourced from Schrödinger’s internal scaffold library (core_library_2014.1-86640.sqlite).

**D. Baseline platforms (BioSolveIT)**

To evaluate the performance of ChemBounce in the context of large-scale chemical space exploration, we compared it with InfiniSee, a scaffold-based virtual screening platform developed by BioSolveIT. InfiniSee enables efficient navigation through ultra-large libraries of synthesizable compounds, facilitating rapid identification of promising candidates by leveraging multi-modal search strategies.

The platform integrates three core modules—FTrees, SpaceMACs, and SpaceLight—each designed to capture different aspects of molecular similarity. FTrees performs fuzzy similarity searches based on topological and pharmacophoric features, enabling the retrieval of non-obvious analogs. SpaceMACs identifies compounds containing specific substructures or motifs of interest, supporting substructure-based scaffold hopping. SpaceLight searches for analogs based on global molecular properties, offering a broader chemical context for hit expansion.

All three modules operate within proprietary enumerated chemical spaces, including REALSpace_48bn_2024-02.space, GalaXi_12bn_2023-03.space, and eXplore_7tr_2022-12.space. The integrated chemical spaces contain over 7 trillion enumerated molecules in total, including 48 billion from REALSpace, 12 billion from GalaXi, and 7 trillion from eXplore.

**D.1. FTrees**

FTrees is a command-line tool optimized for rapid similarity searches over large molecular libraries and combinatorial chemical spaces. It represents molecules as feature trees, where nodes correspond to molecular fragments and edges denote their connectivity. When searching within chemical spaces, FTrees decomposes the query into fragments and identifies similar fragments while preserving the original topological relationships.

In our study, we used FTrees to search within enumerated chemical spaces. The --max-nof-results option was set to 10,000, limiting the number of retrieved hits, and the --min-similarity-threshold was set to 0.5. It is worth noting that the FTrees similarity score does not have a direct one-to-one correspondence with the proportion of atoms or bonds matched; a value of 0.5, for example, does not imply that 50% of the molecule is similar to the query.

**D.2. SpaceMACS**

SpaceMACS is a command-line utility designed for maximum common substructure (MCS) and classical substructure searches within combinatorial chemical spaces of trillion-scale size. Unlike traditional substructure matching, SpaceMACS enables efficient MCS searches in non-enumerated "fragment spaces", allowing the identification of structurally related analogs with shared core motifs.

For our comparison, we employed the MCS similarity search mode (option -t 3) using a Tanimoto-like similarity metric. The number of output hits was limited using --max-nof-results 10000, and the minimum similarity threshold was defined with --min-similarity-threshold 0.5. This configuration ensured the retrieval of compounds with high MCS similarity to the input scaffold.

**D.3. SpaceLight**

SpaceLight performs **2D fingerprint-based similarity searches** across combinatorial chemical spaces. It complements FTrees by focusing on identifying structurally close neighbors using molecular fingerprints (e.g., ECFP and CSFP). While FTrees excels at detecting topologically diverse but functionally similar analogs, SpaceLight is more suited for finding closely related structures in large spaces.

In our analysis, we conducted a 2D similarity search using SpaceLight with a fingerprint-based approach. The search was limited to 10,000 results (--max-nof-results 10000) with a minimum similarity threshold of 0.5 (--min-similarity-threshold 0.5). This allowed for rapid identification of structurally similar analogs to the query compound across billions of enumerated candidates.

**E. Performance indexes**

To evaluate the quality, synthetic accessibility, and drug-likeness of the scaffold-hopped molecules, we employed several performance metrics commonly used in cheminformatics and drug discovery.

1) SAscore

The Synthetic Accessibility score (SAscore) estimates the ease of synthesis of a molecule by combining fragment contributions with a complexity penalty. This metric is widely used in early-stage lead optimization, as the synthetic tractability of candidate molecules affects both cost and feasibility of experimental validation. SAscore values range from 1 (highly accessible) to 10 (highly difficult to synthesize), with lower scores indicating greater synthetic feasibility (Ertl and Schuffenhauer, 2009).

2) QED

The Quantitative Estimate of Drug-likeness (QED) metric quantifies drug-likeness by integrating several key physicochemical descriptors, such as molecular weight, lipophilicity (LogP), and topological polar surface area, based on distributions observed in approved drugs. A QED value ranges from 0 (non-drug-like) to 1 (highly drug-like). This score facilitates early selection of promising candidates by estimating how well a compound conforms to the known profiles of marketed drugs (Bickerton *et al.*, 2012).

3) Physicochemical descriptors (MW, LogP, H-bond donors and acceptors)

We further assessed the following molecular descriptors that correlate with oral bioavailability, based on Lipinski’s Rule of Five (Lipinski *et al.*, 2001).

- Molecular weight (MW): optimal range < 500 Da
- LogP: ideal range < 5 (or MLogP < 4.15)
- Number of hydrogen bond donors (HBD): should not exceed 5
- Number of hydrogen bond acceptors (HBA): should not exceed 10

Compounds violating multiple thresholds are considered more likely to exhibit poor solubility or permeability.

4) Realism probability score based on AnoChem (P_Real_)

To assess the synthetic realism of generated molecules, we employed the
P_Real_ score computed using AnoChem, a deep learning-based framework for distinguishing real-world molecules from model-generated artifacts (Gu *et al.*, 2024). AnoChem comprises an anomaly detection model utilizing ECFP4 fingerprint, along with a real/generated classification model based on XGBoost. The logistic regression (LR)-based ensemble model takes the ECFP4 recovery (L_ECFP4_) from the anomaly detection model, classification result from the classification model (P_Real_,_XGB_), and molecular properties of the chemical structures (QED and MW), and the ensemble model generates the final prediction score, P_Real_, which represents the probability of a generated molecule being a real one (Gu *et al.*, 2024).

**F. Comparative analysis of molecular properties in scaffold hopping**

These figures present a comparative analysis of molecular properties resulting from scaffold hopping using ChemBounce and baseline platforms. The analysis comprises two parts:

- **Panel A (Figures S3-S7):** Comparison of five approved drugs (losartan, gefitinib, fostamatinib, darunavir, and ritonavir) using ChemBounce and five baseline tools (Schrödinger: Ligand-Based Core Hopping, Isosteric Matching; BioSolveIT: FTrees, SpaceMACS, SpaceLight). Molecular property distributions (SAscore, QED, molecular weight, LogP, number of hydrogen bond donors/acceptors, and AnoChem-derived P_Real_) are evaluated to assess the drug-likeness and synthetic realism of generated scaffolds.
- **Panel B (Figures S8-S12):** Performance profiling of ChemBounce under varying scaffold generation parameters. Specifically, we varied:
  - Number of fragment candidates: 1,000 vs. 10,000
  - Tanimoto similarity threshold for fragment replacement: 0.5 vs. 0.7
  - Application of Lipinski’s Rule of Five: with vs. without filter applied

These conditions were designed to assess the trade-off between diversity, similarity, and drug-likeness constraints.


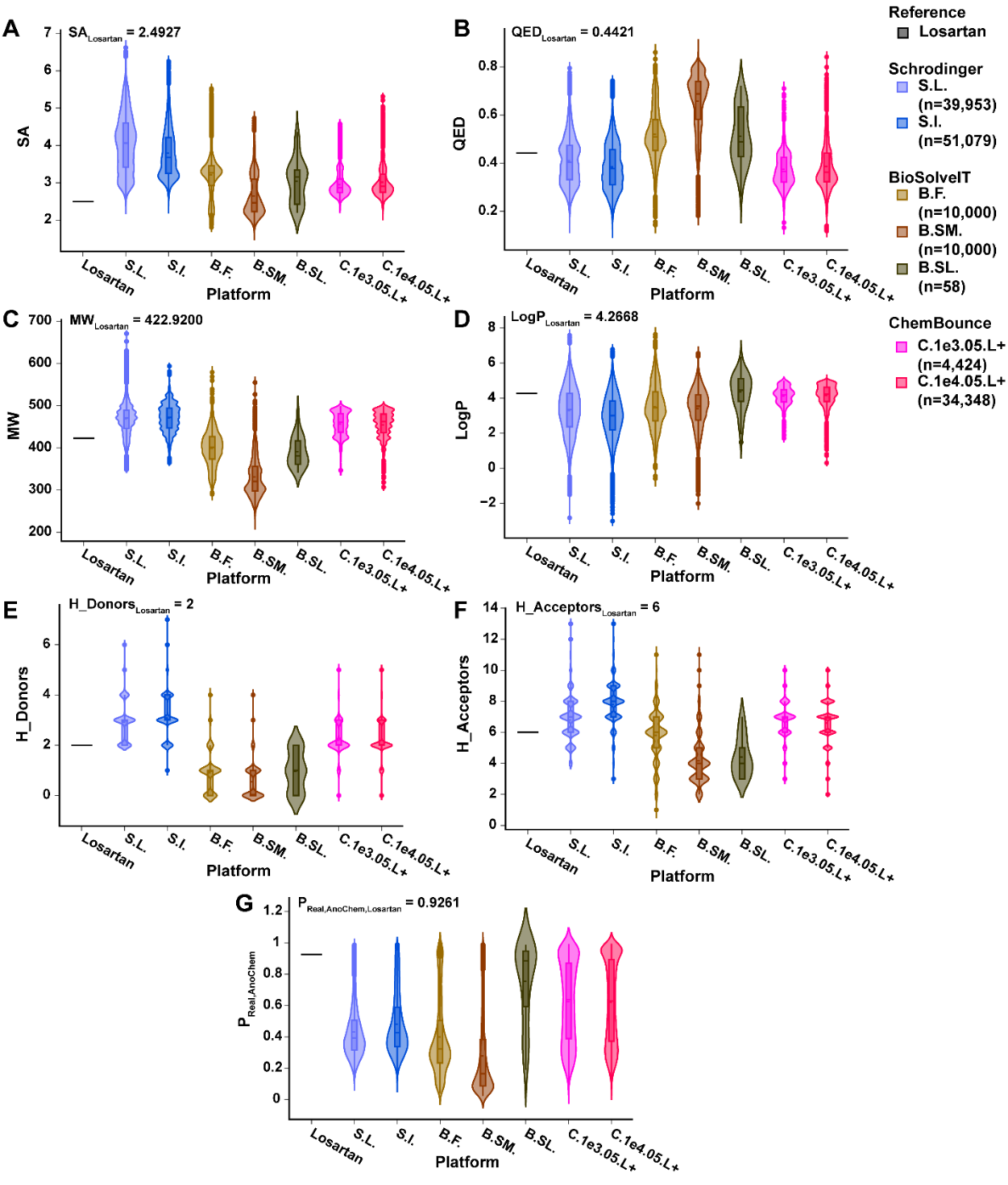


**Figure S3.** **Molecular properties of scaffold-hopping results for baseline platforms and ChemBounce for losartan.** A comparative analysis of molecular properties resulting from scaffold-hopping experiments targeting losartan is presented, based on results obtained from both baseline platforms and ChemBounce. The evaluated properties include (A) synthetic accessibility score (SAscore), (B) quantitative estimate of drug-likeness (QED), (C) molecular weight (MW), (D) LogP, (E) number of hydrogen bond donors, (F) number of hydrogen bond acceptors, and (G) realistic probability obtained using AnoChem (P_Real_). Baseline results were obtained using two Schrödinger modules—Ligand-Based Core Hopping (abbreviated as S.L.) and Isosteric Matching (S.I.)—as well as three tools from BioSolveIT: FTrees (B.F.), SpaceMACS (B.SM.), and SpaceLight (B.SL.). For ChemBounce, two experimental conditions were tested: one with 1,000 fragment candidates (C.1e3.05.L+) and the other with 10,000 candidates (C.1e4.05.L+), both using a Tanimoto similarity threshold of 0.5 and filtering based on Lipinski’s rule of five. Notably, the number of molecules successfully generated by the SpaceLight-based method (B.SL.) was fewer than 100 due to internal constraints and matching limitations.


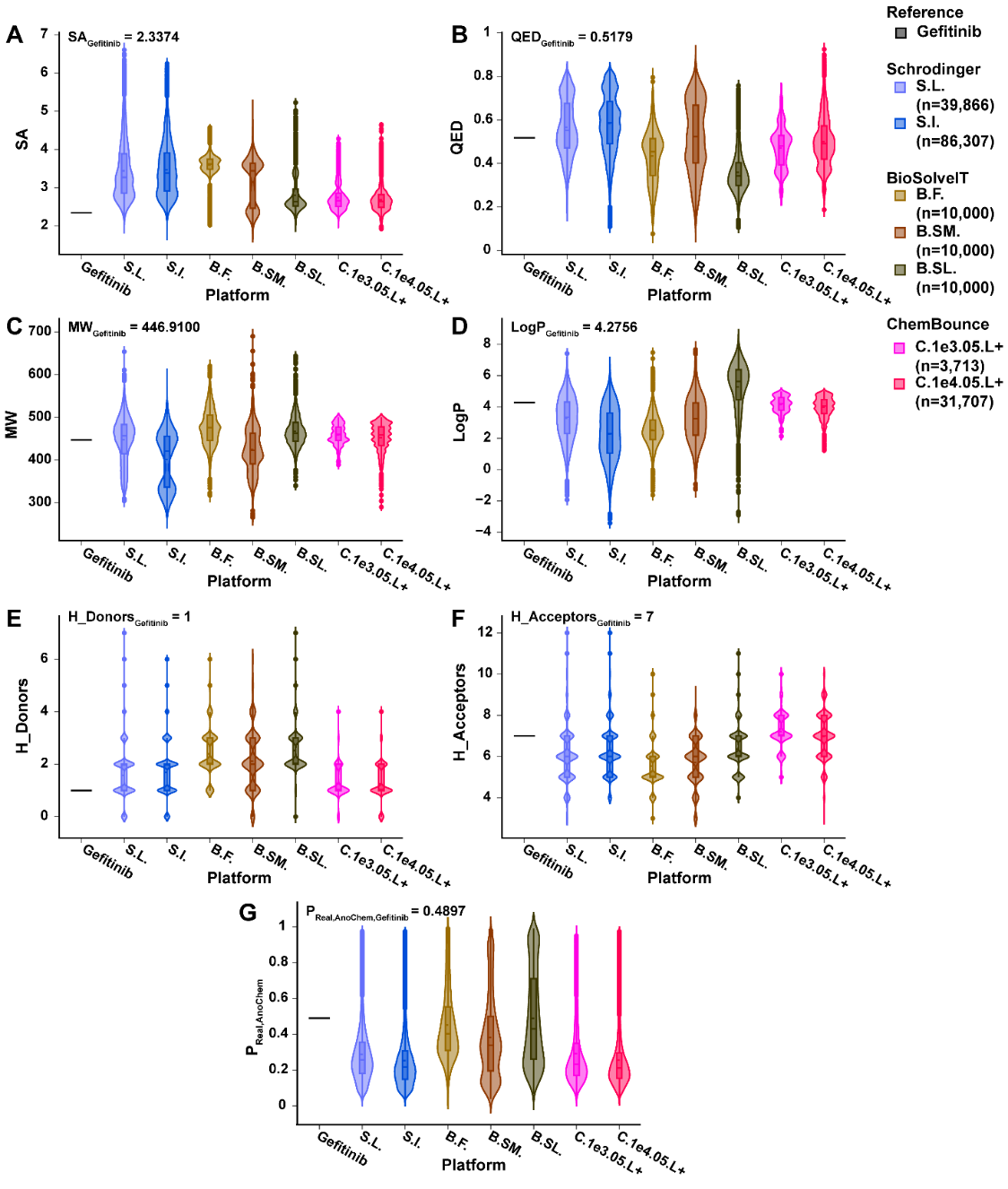


**Figure S4. Molecular properties of scaffold-hopping results for baseline platforms and ChemBounce for gefitinib.** A comparative analysis of molecular properties resulting from scaffold-hopping experiments targeting gefitinib is presented, based on results obtained from both baseline platforms and ChemBounce. The evaluated properties include (A) synthetic accessibility score (SAscore), (B) quantitative estimate of drug-likeness (QED), (C) molecular weight (MW), (D) LogP, (E) number of hydrogen bond donors, (F) number of hydrogen bond acceptors, and (G) realistic probability obtained using AnoChem (P_Real_). Baseline results were obtained using two Schrödinger modules—Ligand-Based Core Hopping (abbreviated as S.L.) and Isosteric Matching (S.I.)—as well as three tools from BioSolveIT: FTrees (B.F.), SpaceMACS (B.SM.), and SpaceLight (B.SL.). For ChemBounce, two experimental conditions were tested: one with 1,000 fragment candidates (C.1e3.05.L+) and the other with 10,000 candidates (C.1e4.05.L+), both using a Tanimoto similarity threshold of 0.5 and filtering based on Lipinski’s rule of five.


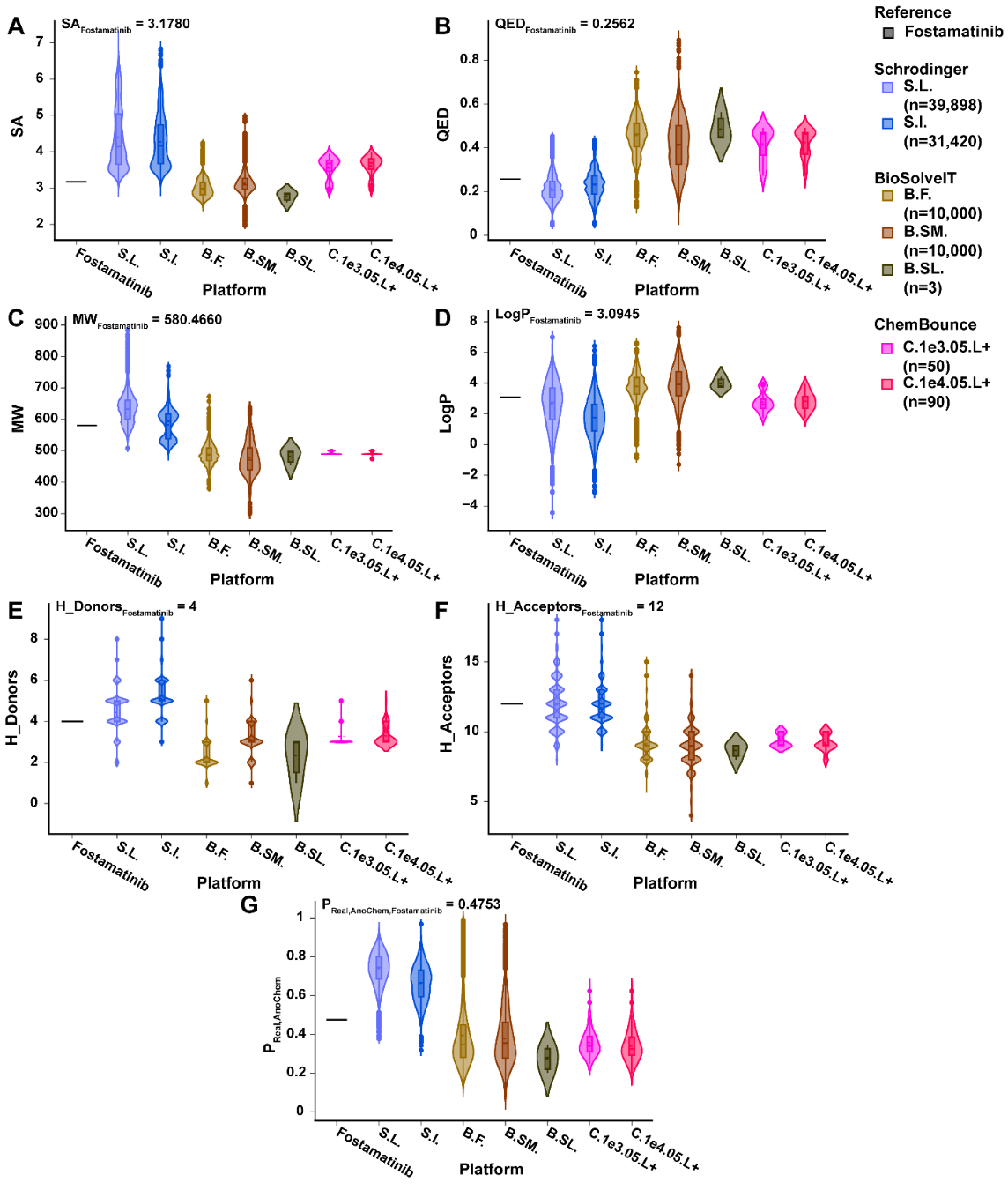


**Figure S5. Molecular properties of scaffold-hopping results for baseline platforms and ChemBounce for fostamatinib.** A comparative analysis of molecular properties resulting from scaffold-hopping experiments targeting fostamatinib is presented, based on results obtained from both baseline platforms and ChemBounce. The evaluated properties include (A) synthetic accessibility score (SAscore), (B) quantitative estimate of drug-likeness (QED), (C) molecular weight (MW), (D) LogP, (E) number of hydrogen bond donors, (F) number of hydrogen bond acceptors, and (G) realistic probability obtained using AnoChem (P_Real_). Baseline results were obtained using two Schrödinger modules—Ligand-Based Core Hopping (abbreviated as S.L.) and Isosteric Matching (S.I.)—as well as three tools from BioSolveIT: FTrees (B.F.), SpaceMACS (B.SM.), and SpaceLight (B.SL.). For ChemBounce, two experimental conditions were tested: one with 1,000 fragment candidates (C.1e3.05.L+) and the other with 10,000 candidates (C.1e4.05.L+), both using a Tanimoto similarity threshold of 0.5 and filtering based on Lipinski’s rule of five. Note that the numbers of generated candidates for B.SL, C.1e3.05.L+, and C.1e4.05.L+ were fewer than 100.


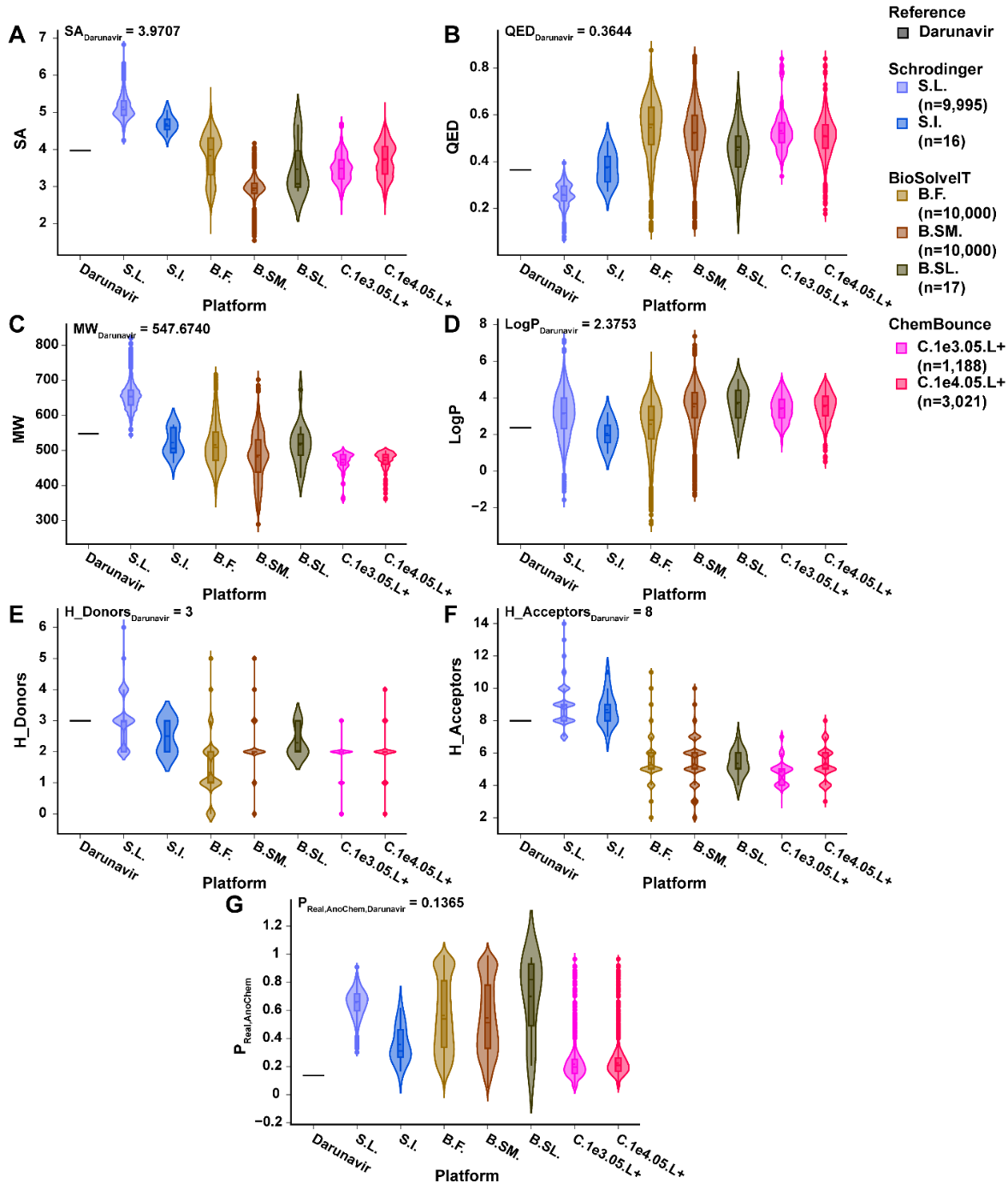


**Figure S6.** **Molecular properties of scaffold-hopping results for baseline platforms and ChemBounce for darunavir.** A comparative analysis of molecular properties resulting from scaffold-hopping experiments targeting darunavir is presented, based on results obtained from both baseline platforms and ChemBounce. The evaluated properties include (A) synthetic accessibility score (SAscore), (B) quantitative estimate of drug-likeness (QED), (C) molecular weight (MW), (D) LogP, (E) number of hydrogen bond donors, (F) number of hydrogen bond acceptors, and (G) realistic probability obtained using AnoChem (P_Real_). Baseline results were obtained using two Schrödinger modules—Ligand-Based Core Hopping (abbreviated as S.L.) and Isosteric Matching (S.I.)—as well as three tools from BioSolveIT: FTrees (B.F.), SpaceMACS (B.SM.), and SpaceLight (B.SL.). For ChemBounce, two experimental conditions were tested: one with 1,000 fragment candidates (C.1e3.05.L+) and the other with 10,000 candidates (C.1e4.05.L+), both using a Tanimoto similarity threshold of 0.5 and filtering based on Lipinski’s rule of five. Note that the numbers of generated candidates for S.I. and B.SL. were fewer than 100.


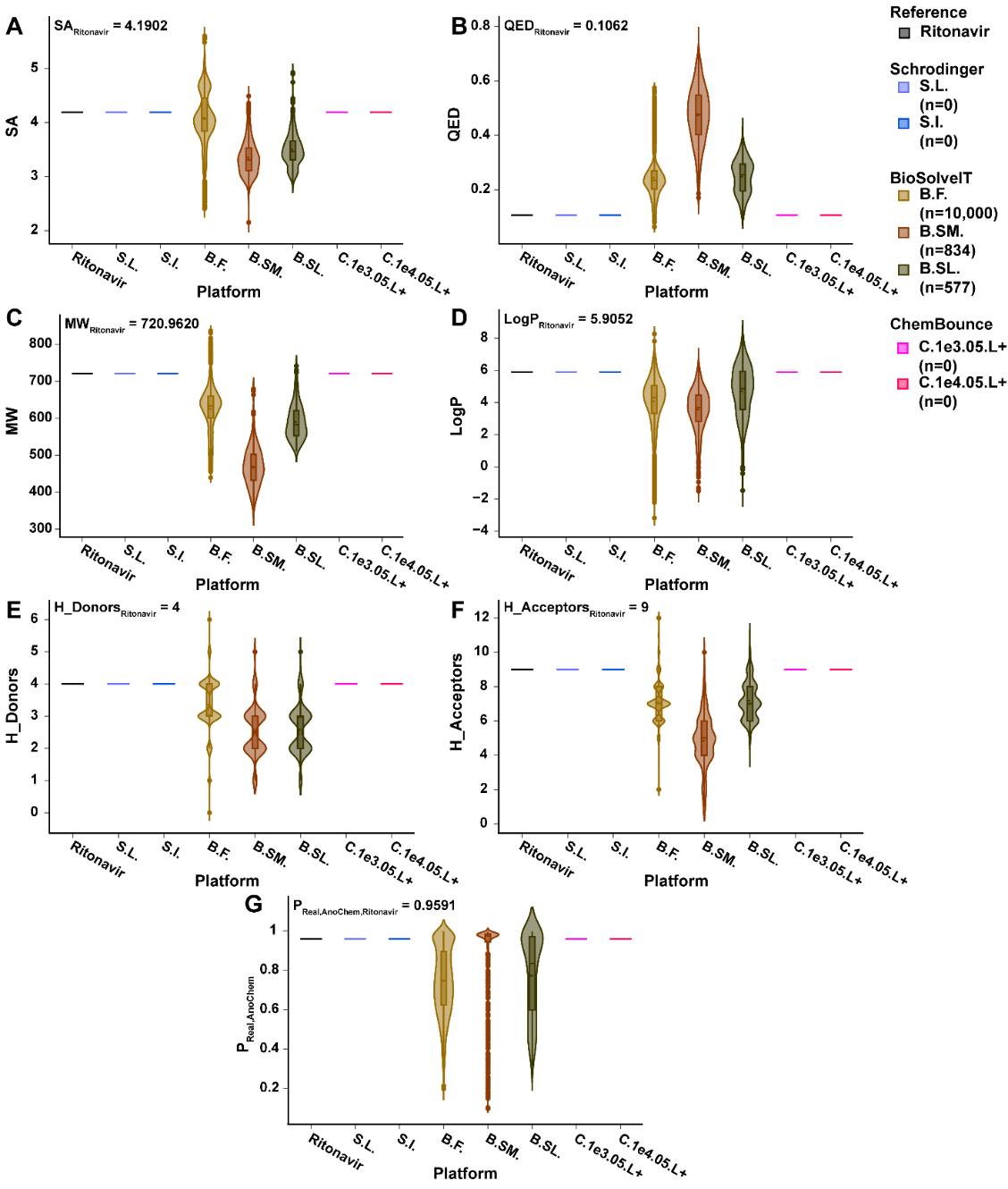


**Figure S7.** **Molecular properties of scaffold-hopping results for baseline platforms and ChemBounce for ritonavir.** A comparative analysis of molecular properties resulting from scaffold-hopping experiments targeting ritonavir is presented, based on results obtained from both baseline platforms and ChemBounce. The evaluated properties include (A) synthetic accessibility score (SAscore), (B) quantitative estimate of drug-likeness (QED), (C) molecular weight (MW), (D) LogP, (E) number of hydrogen bond donors, (F) number of hydrogen bond acceptors, and (G) realistic probability (P_Real_) obtained using AnoChem. Baseline results were obtained using two Schrödinger modules—Ligand-Based Core Hopping (abbreviated as S.L.) and Isosteric Matching (S.I.)—as well as three tools from BioSolveIT: FTrees (B.F.), SpaceMACS (B.SM.), and SpaceLight (B.SL.). For ChemBounce, two experimental conditions were tested: one with 1,000 fragment candidates (C.1e3.05.L+) and the other with 10,000 candidates (C.1e4.05.L+), both using a Tanimoto similarity threshold of 0.5 and filtering based on Lipinski’s rule of five. Note that no candidates were generated under the C.1e3.05.L+ and C.1e4.05.L+ conditions. In the case of ritonavir, due to its large molecular size, the number of possible scaffold definitions exceeded the practical limits of the Schrödinger-based methods, so meaningful results could not be obtained; therefore, data from these methods were excluded from the analysis.


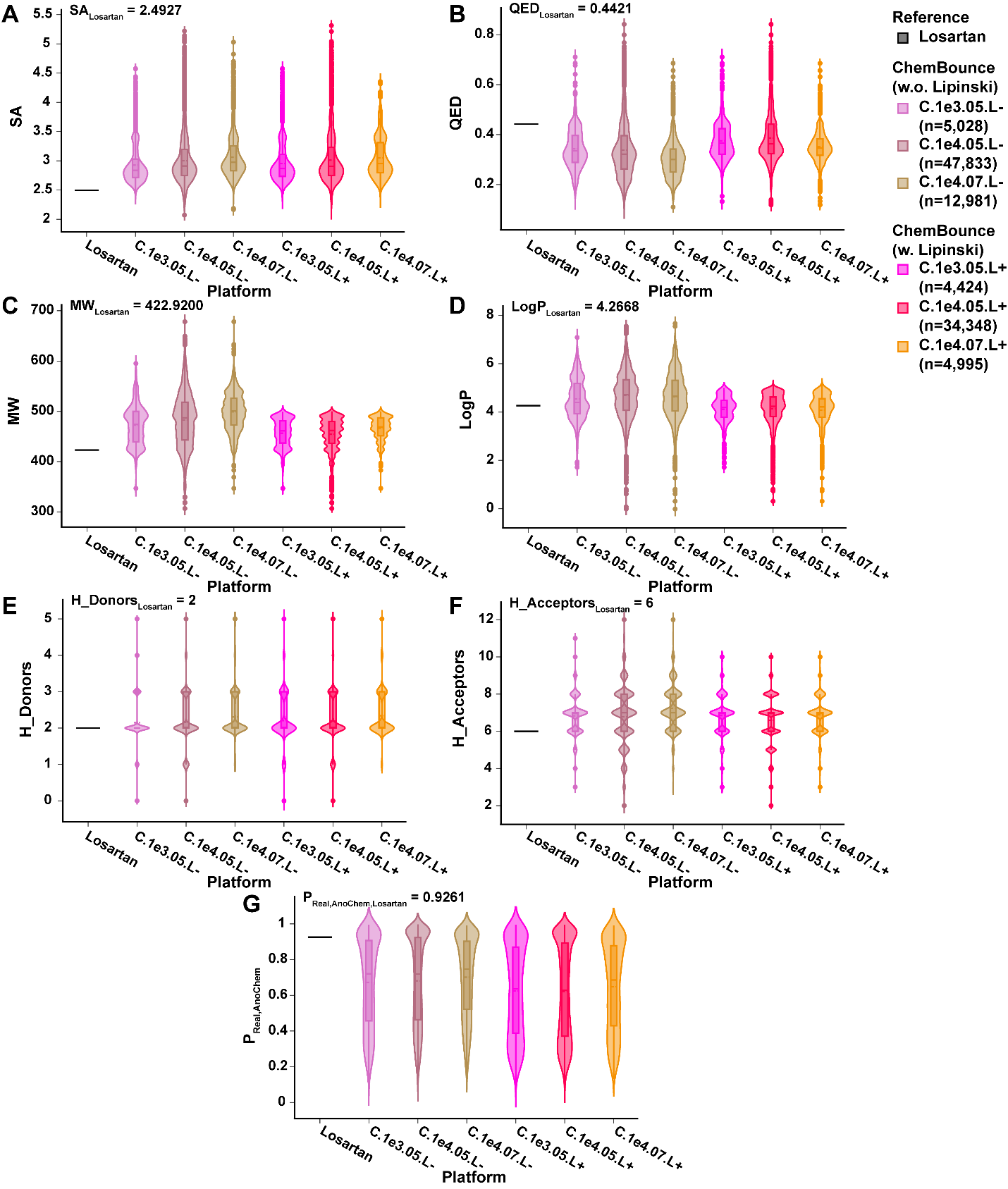


**Figure S8. Molecular properties of scaffold-hopping results for losartan under various ChemBounce conditions.** The evaluated properties include (A) synthetic accessibility score (SAscore), (B) quantitative estimate of drug-likeness (QED), (C) molecular weight (MW), (D) LogP, (E) number of hydrogen bond donors, (F) number of hydrogen bond acceptors, and (G) realistic probability (P_Real_) as predicted by AnoChem. ChemBounce conditions were varied by altering the number of fragment candidates (1,000 vs. 10,000), the Tanimoto similarity threshold (0.5 vs. 0.7), and the application of Lipinski’s rule of five. Each panel corresponds to one molecular property and summarizes the distribution of values under the respective conditions.


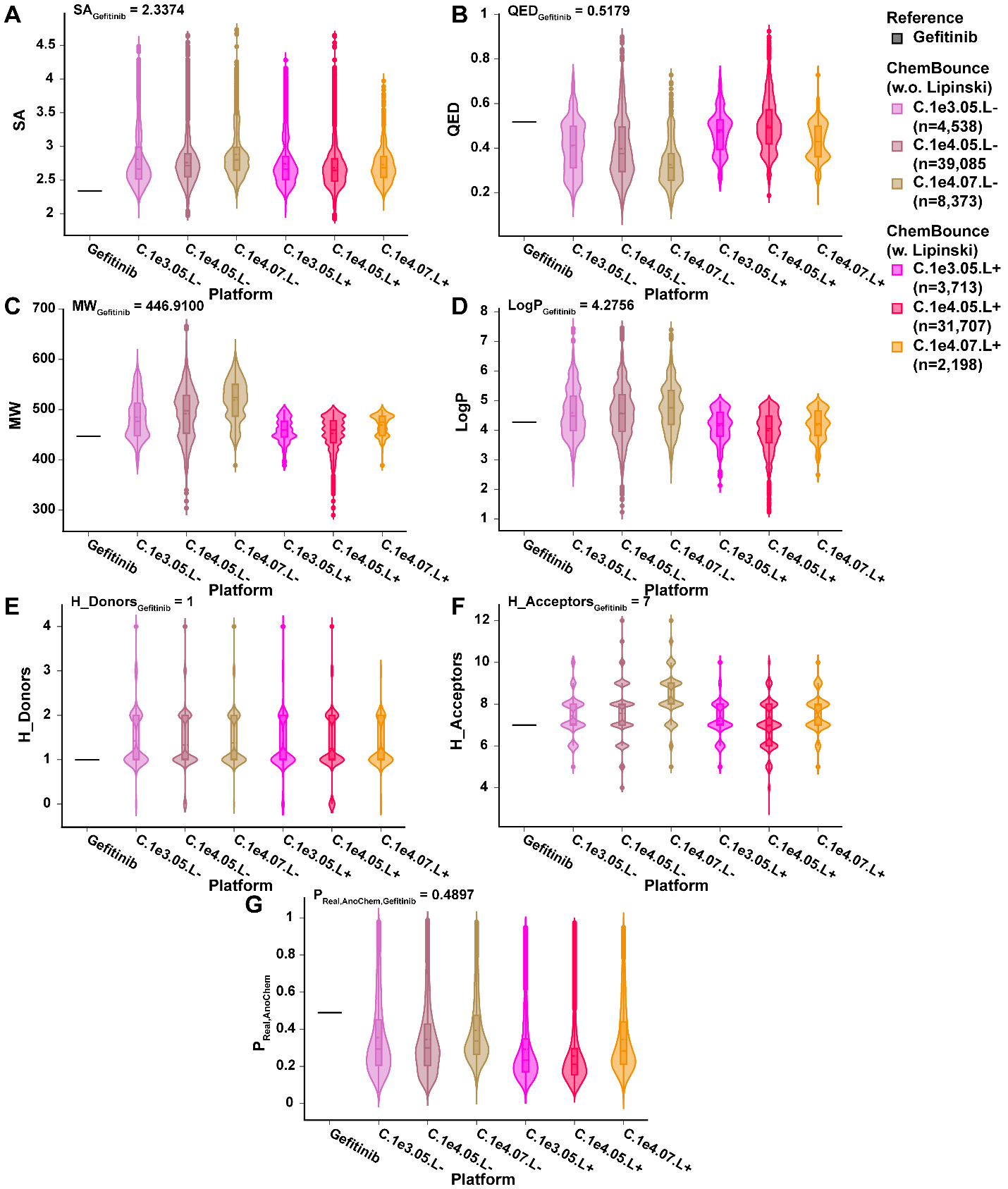


**Figure S9. Molecular properties of scaffold-hopping results for gefitinib under various ChemBounce conditions.** The evaluated properties include (A) synthetic accessibility score (SAscore), (B) quantitative estimate of drug-likeness (QED), (C) molecular weight (MW), (D) LogP, (E) number of hydrogen bond donors, (F) number of hydrogen bond acceptors, and (G) realistic probability (P_Real_) as predicted by AnoChem. ChemBounce conditions were varied by altering the number of fragment candidates (1,000 vs. 10,000), the Tanimoto similarity threshold (0.5 vs. 0.7), and the application of Lipinski’s rule of five. Each panel corresponds to one molecular property and summarizes the distribution of values under the respective conditions.


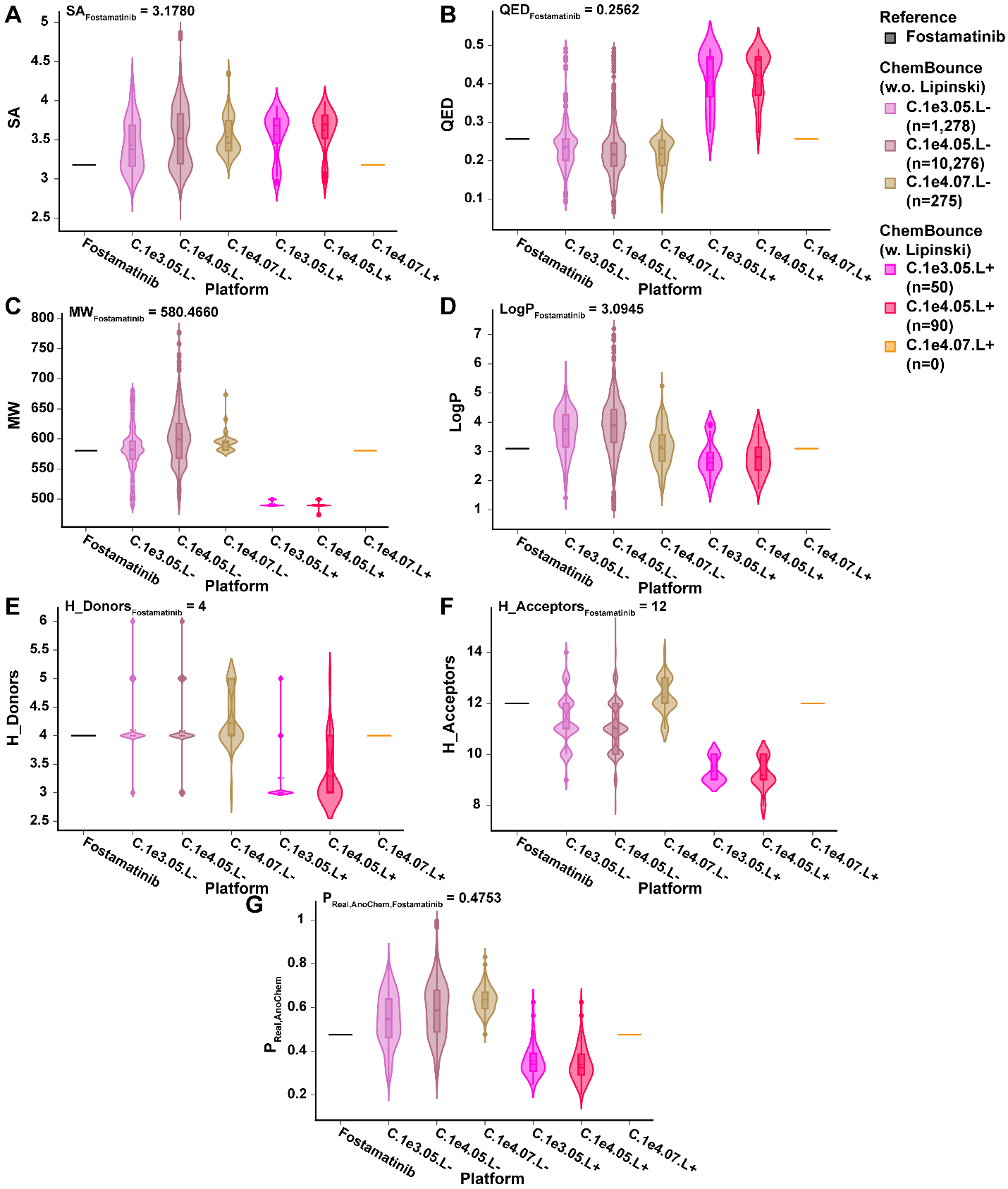


**Figure S10. Molecular properties of scaffold-hopping results for fostamatinib under various ChemBounce conditions.** The evaluated properties include (A) synthetic accessibility score (SAscore), (B) quantitative estimate of drug-likeness (QED), (C) molecular weight (MW), (D) LogP, (E) number of hydrogen bond donors, (F) number of hydrogen bond acceptors, and (G) realistic probability (P_Real_) as predicted by AnoChem. ChemBounce conditions were varied by altering the number of fragment candidates (1,000 vs. 10,000), the Tanimoto similarity threshold (0.5 vs. 0.7), and the application of Lipinski’s rule of five. Each panel corresponds to one molecular property and summarizes the distribution of values under the respective conditions. When Lipinski’s rule of five was applied during scaffold hopping for fostamatinib, ChemBounce identified few candidate molecules, primarily due to constraints on the number of hydrogen bond acceptors and molecular weight.


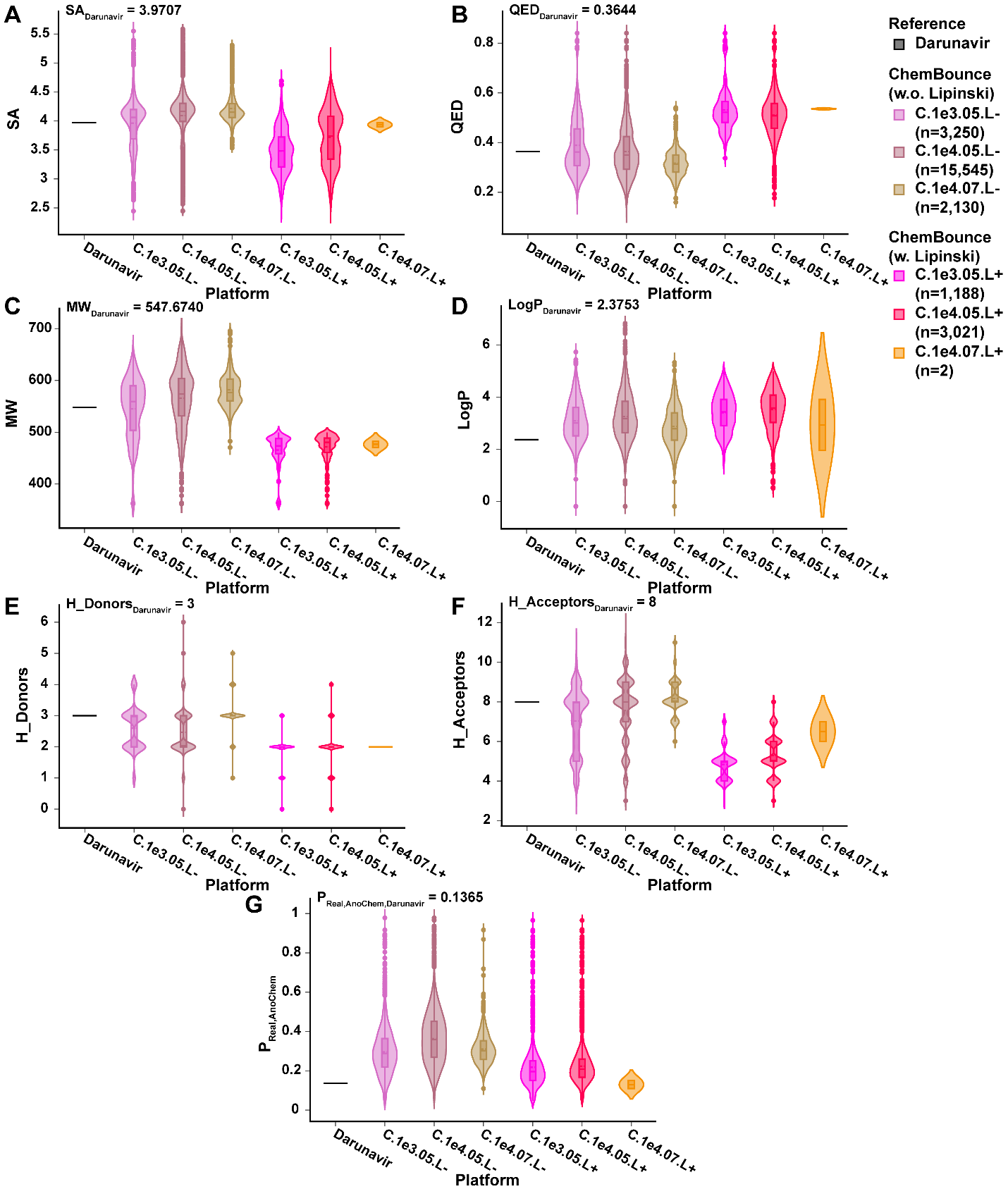


**Figure S11. Molecular properties of scaffold-hopping results for darunavir under various ChemBounce conditions.** The evaluated properties include (A) synthetic accessibility score (SAscore), (B) quantitative estimate of drug-likeness (QED), (C) molecular weight (MW), (D) LogP, (E) number of hydrogen bond donors, (F) number of hydrogen bond acceptors, and (G) realistic probability (P_Real_) as predicted by AnoChem. ChemBounce conditions were varied by altering the number of fragment candidates (1,000 vs. 10,000), the Tanimoto similarity threshold (0.5 vs. 0.7), and the application of Lipinski’s rule of five. Each panel corresponds to one molecular property and summarizes the distribution of values under the respective conditions.


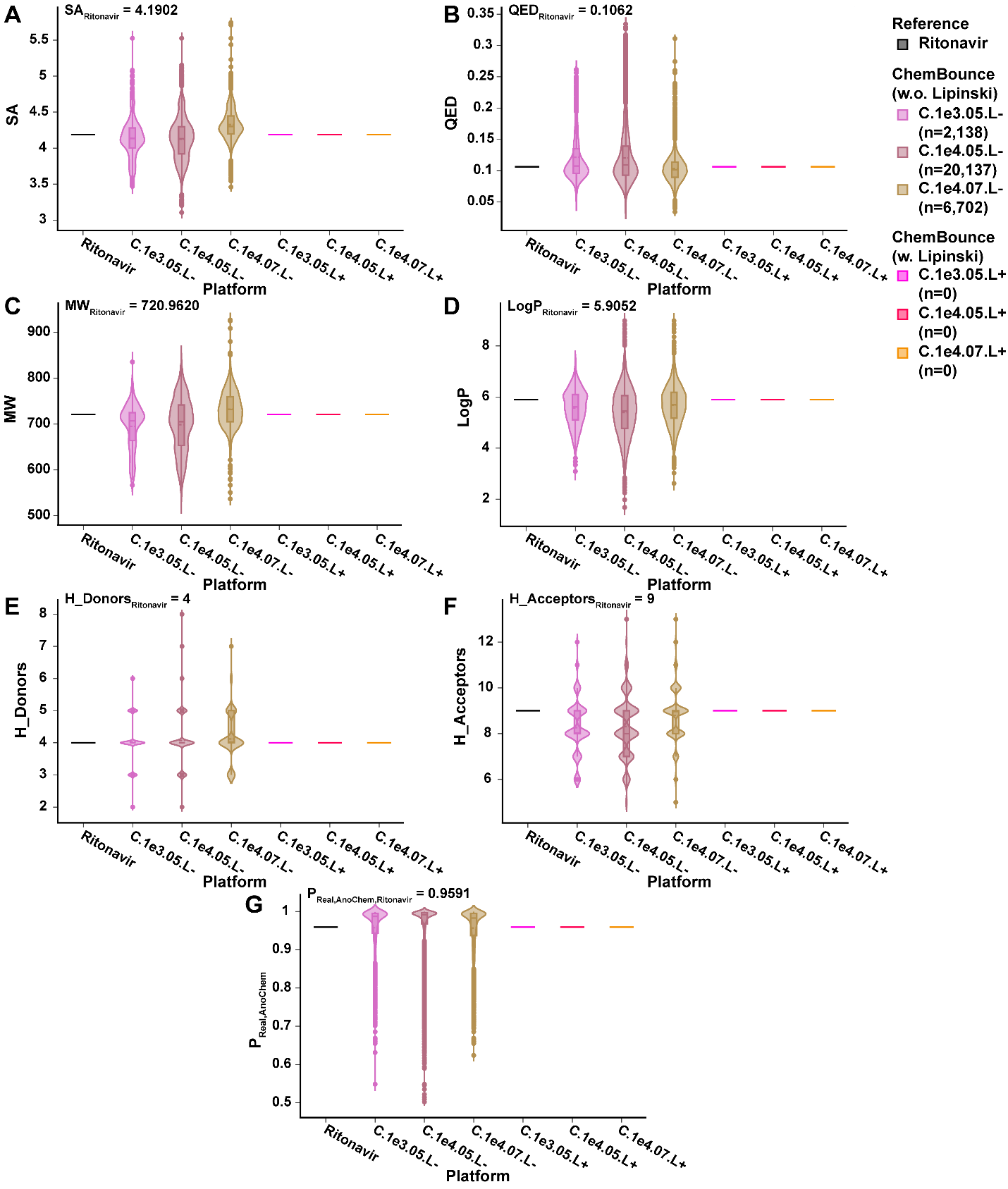


**Figure S12. Molecular properties of scaffold-hopping results for ritonavir under various ChemBounce conditions.** The evaluated properties include (A) synthetic accessibility score (SAscore), (B) quantitative estimate of drug-likeness (QED), (C) molecular weight (MW), (D) LogP, (E) number of hydrogen bond donors, (F) number of hydrogen bond acceptors, and (G) realistic probability (P_Real_) as predicted by AnoChem. ChemBounce conditions were varied by altering the number of fragment candidates (1,000 vs. 10,000), the Tanimoto similarity threshold (0.5 vs. 0.7), and the application of Lipinski’s rule of five. Each panel corresponds to one molecular property and summarizes the distribution of values under the respective conditions. When Lipinski’s rule of five was applied during scaffold hopping for ritonavir, ChemBounce failed to generate any candidate molecules, primarily due to the compound’s high molecular weight and, potentially, its elevated LogP.

**Table S1. A comprehensive failure-case reference sheet**

|  | **SMILES** | **Cause of the problem** | **Error log message** |
| --- | --- | --- | --- |
| **Case_1** | C1CC([R])CCC1C(=O)NCC2=CC=CC=C2 | Invalid atomic symbol ‘([R])’ not present in the periodic table | utils.SmilesInputError: Invalid SMILES input for rdkit |
| **Case_2** | B1(OB(OB(O1)C=C)C=C)C=C.C1=CC=NC=C1 | Multi-component system containing mixture/salt forms | Multi-component (mixture or salt form) detected - please separate into individual compounds |
| **Case_3** | CCN(CC)CC.[ClH] | Salt form with ionic components requiring separation | Multi-component (mixture or salt form) detected - please separate into individual compounds |
| **Case_4** | O=C(NC1=CC=CC=C1)C2=CC=A=CC=C2 | Invalid atomic symbol ‘A’ not present in the periodic table | utils.SmilesInputError: Invalid SMILES input for rdkit |
| **Case_5** | O=N([O-])C1=C(CN=C1NCCSCc2ncccc2)Cc3ccccc3 | Invalid valence state and improper atomic connectivity | utils.SmilesInputError: Invalid SMILES input for rdkit |
| **Case_6** | FC1=CC(C2CC2)=CC(C=CN3C4=CC=CC=C4C(=C1C3=O | Malformed SMILES syntax with incorrect ring closure notation | utils.SmilesInputError: Invalid SMILES input for rdkit |

**Table S2. Performance validation of ChemBounce across diverse molecular types**

| **Compound** | **Molecule type** | **Molecular weight (Da)** | **Number of detected scaffolds** | **Processing time** | **SMILES** |
| --- | --- | --- | --- | --- | --- |
| Kyprolis | Peptide | 719.9 | 7 | 1 min 22 s | CC(C)C[C@@H](C(=O)[C@]1(CO1)C)NC(=O)[C@H](CC2=CC=CC=C2)NC(=O)[C@H](CC(C)C)NC(=O)[C@H](CCC3=CC=CC=C3)NC(=O)CN4CCOCC4 |
| Trofinetide | Peptide | 315.32 | 1 | 4 s | C[C@]1(CCCN1C(=O)CN)C(=O)N[C@@H](CCC(=O)O)C(=O)O |
| Mounjaro | Peptide | 4813 | 16 | 2 min 57 s | CC[C@H](C)[C@@H](C(=O)N[C@@H](C)C(=O)N[C@@H](CCC(=O)N)C(=O)N[C@@H](CCCCNC(=O)COCCOCCNC(=O)COCCOCCNC(=O)CC[C@@H](C(=O)O)NC(=O)CCCCCCCCCCCCCCCCCCC(=O)O)C(=O)N[C@@H](C)C(=O)N[C@@H](CC1=CC=CC=C1)C(=O)N[C@@H](C(C)C)C(=O)N[C@@H](CCC(=O)N)C(=O)N[C@@H](CC2=CNC3=CC=CC=C32)C(=O)N[C@@H](CC(C)C)C(=O)N[C@@H]([C@@H](C)CC)C(=O)N[C@@H](C)C(=O)NCC(=O)NCC(=O)N4CCC[C@H]4C(=O)N[C@@H](CO)C(=O)N[C@@H](CO)C(=O)NCC(=O)N[C@@H](C)C(=O)N5CCC[C@H]5C(=O)N6CCC[C@H]6C(=O)N7CCC[C@H]7C(=O)N[C@@H](CO)C(=O)N)NC(=O)[C@H](CCCCN)NC(=O)[C@H](CC(=O)O)NC(=O)[C@H](CC(C)C)NC(=O)C(C)(C)NC(=O)[C@H]([C@@H](C)CC)NC(=O)[C@H](CO)NC(=O)[C@H](CC8=CC=C(C=C8)O)NC(=O)[C@H](CC(=O)O)NC(=O)[C@H](CO)NC(=O)[C@H]([C@@H](C)O)NC(=O)[C@H](CC9=CC=CC=C9)NC(=O)[C@H]([C@@H](C)O)NC(=O)CNC(=O)[C@H](CCC(=O)O)NC(=O)C(C)(C)NC(=O)[C@H](CC1=CC=C(C=C1)O)N |
| Pasireotide | Macrocyclic peptide | 1047.2 | 11 | 34 s | C1[C@H](CN2[C@@H]1C(=O)N[C@H](C(=O)N[C@@H](C(=O)N[C@H](C(=O)N[C@H](C(=O)N[C@H](C2=O)CC3=CC=CC=C3)CC4=CC=C(C=C4)OCC5=CC=CC=C5)CCCCN)CC6=CNC7=CC=CC=C76)C8=CC=CC=C8)OC(=O)NCCN |
| Motixafortide | Macrocyclic peptide | 2159.5 | 9 | 8 min 24 s | C1C[C@H]2C(=O)N[C@H](C(=O)N[C@H](C(=O)N[C@H](C(=O)N[C@@H](CSSC[C@@H](C(=O)N[C@H](C(=O)N[C@H](C(=O)N[C@H](C(=O)N[C@H](C(=O)N2C1)CCCCN)CCCCN)CCCNC(=O)N)CC3=CC=C(C=C3)O)NC(=O)[C@H](CC4=CC5=CC=CC=C5C=C4)NC(=O)[C@H](CCCNC(=N)N)NC(=O)[C@H](CCCNC(=N)N)NC(=O)C6=CC=C(C=C6)F)C(=O)N[C@@H](CCCNC(=N)N)C(=O)N)CCCNC(=O)N)CCCNC(=N)N)CC7=CC=C(C=C7)O |
| Celecoxib | Small molecule | 381.4 | 5 | 2 min 26 s | CC1=CC=C(C=C1)C2=CC(=NN2C3=CC=C(C=C3)S(=O)(=O)N)C(F)(F)F |
| Rimonabant | Small molecule | 463.8 | 7 | 6 min 19 s | CC1=C(N(N=C1C(=O)NN2CCCCC2)C3=C(C=C(C=C3)Cl)Cl)C4=CC=C(C=C4)Cl |
| Lapatinib | Small molecule | 581.1 | 6 | 4 min 27 s | CS(=O)(=O)CCNCC1=CC=C(O1)C2=CC3=C(C=C2)N=CN=C3NC4=CC(=C(C=C4)OCC5=CC(=CC=C5)F)Cl |
| Trametinib | small molecule | 615.4 | 7 | 13 min 6 s | CC1=C2C(=C(N(C1=O)C)NC3=C(C=C(C=C3)I)F)C(=O)N(C(=O)N2C4=CC=CC(=C4)NC(=O)C)C5CC5 |
| Venetoclax | small molecule | 868.4 | 13 | 21 min 39 s | CC1(CCC(=C(C1)C2=CC=C(C=C2)Cl)CN3CCN(CC3)C4=CC(=C(C=C4)C(=O)NS(=O)(=O)C5=CC(=C(C=C5)NCC6CCOCC6)[N+](=O)[O-])OC7=CN=C8C(=C7)C=CN8)C |

**References**

Bickerton,G.R. *et al.* (2012) Quantifying the chemical beauty of drugs. *Nat. Chem.*, **4**, 90-98.

Degen,J. *et al.* (2008) On the art of compiling and using 'drug-like' chemical fragment spaces. *ChemMedChem*, **3**, 1503-1507.

Ertl,P. and Schuffenhauer,A. (2009) Estimation of synthetic accessibility score of drug-like molecules based on molecular complexity and fragment contributions. *J. Cheminform.*, **1**, 8.

Gu,C. *et al.* (2024) Anochem: Prediction of chemical structural abnormalities based on machine learning models. *Comput. Struct. Biotechnol. J.*, **23**, 2116-2121.

Lipinski,C.A. *et al.* (2001) Experimental and computational approaches to estimate solubility and permeability in drug discovery and development settings. *Adv. Drug Deliv. Rev.*, **46**, 3-26.

Wilkens,S.J., Janes,J. and Su,A.I. (2005) Hiers: Hierarchical scaffold clustering using topological chemical graphs. *J. Med. Chem.*, **48**, 3182-3193.
